# Progress in Photo-Responsive Polypeptide Derived Nano-Assemblies

**DOI:** 10.3390/mi9060296

**Published:** 2018-06-13

**Authors:** Lu Yang, Houliang Tang, Hao Sun

**Affiliations:** 1 Department of Chemistry, University of Florida, Gainesville, FL 32611, USA; yanglulucia@chem.ufl.edu; 2 Department of Chemistry, Southern Methodist University, Dallas, TX 75275, USA; houliangt@smu.edu

**Keywords:** stimuli-responsive polymers, synthetic polypeptide, photo-sensitive, self-assembly, morphological transformation

## Abstract

Stimuli-responsive polymeric materials have attracted significant attention in a variety of high-value-added and industrial applications during the past decade. Among various stimuli, light is of particular interest as a stimulus because of its unique advantages, such as precisely spatiotemporal control, mild conditions, ease of use, and tunability. In recent years, a lot of effort towards the synthesis of a biocompatible and biodegradable polypeptide has resulted in many examples of photo-responsive nanoparticles. Depending on the specific photochemistry, those polypeptide derived nano-assemblies are capable of crosslinking, disassembling, or morphing into other shapes upon light irradiation. In this review, we aim to assess the current state of photo-responsive polypeptide based nanomaterials. Firstly, those ‘smart’ nanomaterials will be categorized by their photo-triggered events (i.e., crosslinking, degradation, and isomerization), which are inherently governed by photo-sensitive functionalities, including *O*-nitrobenzyl, coumarin, azobenzene, cinnamyl, and spiropyran. In addition, the properties and applications of those polypeptide nanomaterials will be highlighted as well. Finally, the current challenges and future directions of this subject will be evaluated.

## 1. Introduction

**Scheme 1:**
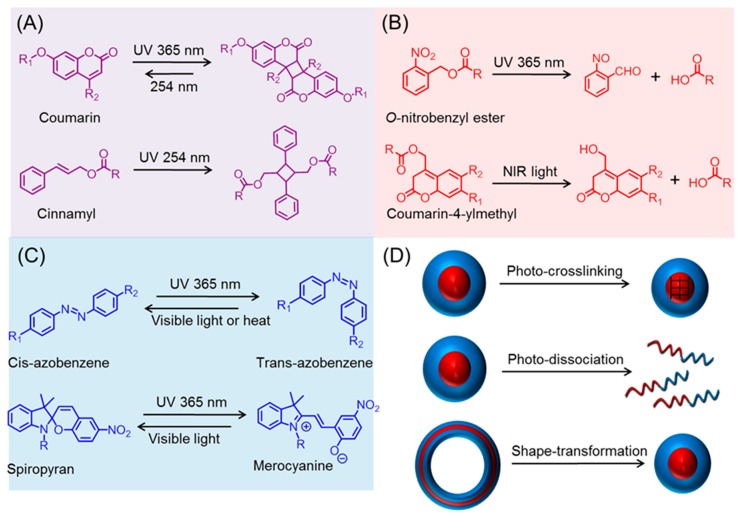
Photo-chemistry of various functional groups. (**A**) Ultraviolet (UV)-induced dimerization; (**B**) UV or near infrared (NIR) promoted cleavage; (**C**) reversible isomerization by UV and visible light; and (**D**) photo-triggered metamorphosis of polypeptide derived nano-objects.

Stimuli-responsive or ‘Smart’ polymers are capable of changing their physical and/or chemical properties upon receiving external triggers, such as temperature, pH, redox, mechanical forces, and light [1,2,3,4,5,6,7,8,9]. These tailor-made polymers are receiving significant interest in the fields of drug delivery, biosensor, tissue engineering, coatings, and self-healing materials [10,11,12,13,14]. In particular, light has recently garnered tremendous attention as a stimulus, as it can be not only triggered remotely but also provides spatiotemporal control [15,16,17,18,19,20,21,22,23]. Moreover, irradiation parameters, including wavelength, power, and time, can be easily tuned to fit the system (e.g., on-demand and controllable drug release rate) [24,25,26,27]. Typically, the ability of smart polymers to respond to light stems from the incorporation of photo-sensitive chemical structures [28,29,30]. Those moieties can be classified into three general categories based on their specific photo-chemistry (Scheme 1A–C). In the first category, represented by cinnamyl and coumarin, photo-induced dimerization of those groups takes place upon irradiation at a certain wavelength, while the dimer can undergo a reversal reaction at another wavelength with a higher energy (i.e., shorter wavelength). The second (e.g., *O*-nitrobenzyl) involves irreversible photo-triggered degradation, which can liberate the unprotected functionality, leading to a dramatic change in solvability. The last subset includes functional groups, such as azobenzene and spiropyran, which are capable of reversibly isomerizing under different wavelengths. By taking advantage of the above-mentioned photo-chemistry, various light-triggered morphological transformations (e.g., crosslinking, dissociation, and shape change) of polypeptide nano-assemblies have been achieved (Scheme 1D).

Inspired by natural protein, synthetic polypeptides or poly(amino acids) based nanomaterials are receiving increasing interest in the field of polymer science because of their inherent biocompatibility and biodegradability [31,32]. Furthermore, synthetic polypeptides have exhibited their unique ability to form higher order secondary structures, including α-helix, β-sheet, and β-turn, thanks to non-covalent interactions (i.e., hydrogen bonds, pi–pi stacking, and hydrophobic interaction) between amino acids side chains [33,34]. Those non-covalent interactions are highly sensitive to local environments, such as temperature, pH, the presence and concentration of reducing agent, ionic strength, and even light. A small change in the local environment could have a noticeable impact on non-covalent interactions, resulting in the transformation of secondary structures and concomitant change in bio-activity and function of polypeptides [35].

**Scheme 2:**
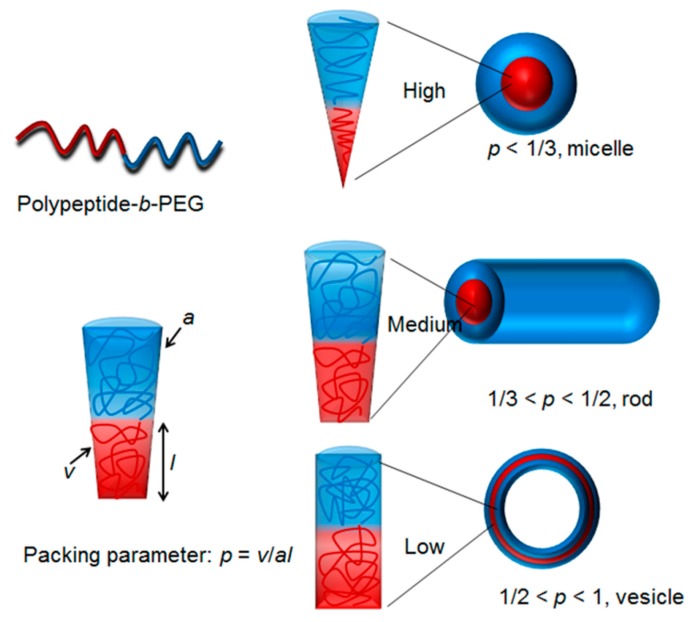
The shapes of polypeptide derived nano-objects are dictated by critical packing parameters [65]. Diblock copolymer polypeptide-*b*-PEG exemplifies amphiphilic polypeptide based copolymers.

The rapid development of polymerization methodology has empowered polymer chemists with the ability to easily prepare unique polypeptides with diverse architecture and functionalities [36,37,38,39]. Numerous polypeptides have been successfully prepared via various living polymerization approaches, such as ring-opening polymerization of *N*-carboxyanhydrides (NCA) [40,41,42,43], reversible addition-fragmentation polymerization [38], atom transfer radical polymerization [44,45], and ring-opening metathesis polymerization [46,47,48,49,50]. In a typical case, living or controlled polymerization techniques are capable of producing polymers with precise chain lengths, excellent functionalities tolerance, and narrow polydispersity [51,52,53,54,55]. In addition, complicated architectures, such as block, cyclic, brush, and star, which were previously inaccessible, can now easily be made via living polymerization techniques [56,57,58,59,60,61,62,63,64]. Owing to those features arising from the living polymerizations (vide supra), one can design and tune the hydrophobic to hydrophilic balance, which dictates the critical packing parameters and give rise to nanoparticles with predictable morphologies (Scheme 2) [65].

With the recent success of light-responsive amphiphilic polypeptides in nanotechnology and nanomedicine, we believe it is necessary to assess the current state of those smart nanomaterials. In this review, the main focus will be placed on the photo-chemistry of various light-sensitive functional groups that are incorporated into the polypeptide nanoparticles. Furthermore, we will discuss the influence on the size or morphologies of nano-assemblies as a consequence of light treatment and how this may assist the prediction of potential applications of those materials. Recent examples of photo-responsive polypeptide deriving nanoparticles are summarized in Table 1. Finally, we believe it is crucial to evaluate the current challenges and future directions of this field.

## 2. Photo-Chemistry of Light-Responsive Polypeptide Nanoparticles

### 2.1. Photo-Crosslinkable Nanoparticles

Photo-dimerizable or crosslinkable groups including cinnamic, coumarin, and anthracene can undergo a crosslinking reaction via [2+2] cycloaddition of the carbon-carbon double bonds after UV-irradiation [79,80,81]. They have been mainly utilized for the photo-crosslinking of micelles, leading to micelles or nanogels with enhanced colloidal stability, even in very dilute condition [82,83,84]. Compared with traditional crosslinking methods such as ‘click’ chemistry and carbodiimide coupling, the photo-crosslinking approach is relatively inexpensive, rapid, and highly efficient at room temperature. Furthermore, no byproduct is generated during the photo-dimerization process, rendering a final product with a high purity [85].

**Figure 1:**
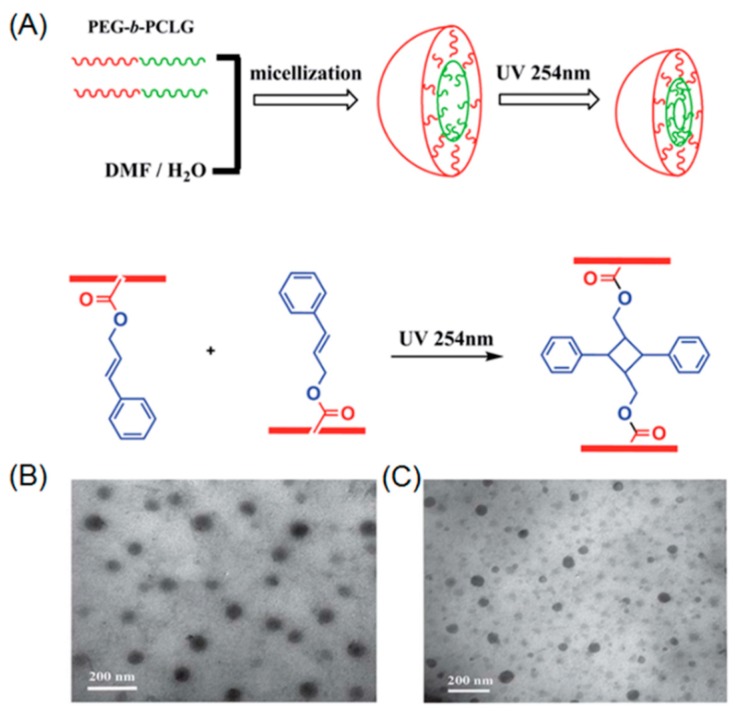
(**A**) Self-assembly of PEG-*b*-polypeptide and subsequent photo-induced core-crosslinking; (**B**) Transmission electron microscopy （TEM） image before UV irradiation; and (**C**) TEM image after UV irradiation. Reproduced with permission from [67].

Chen et al. demonstrated the first example of photo-crosslinkable polypeptide based micelle [66]. In their work, diblock copolymer poly(ethylene glycol)-*b*-poly(l-glutamic acid) was synthesized by ring-opening polymerization (ROP) of l-glutamate-NCA monomer in the presence of PEG-amine macroinitiator. The resulting diblock copolymer further underwent deprotection and subsequent modification with cinnamyl alcohol, yielding amphiphilic PEG-*b*-polypeptide, containing pendent cinnamyl functionalities. A core-shell micellar structure was formed by the self-assembly of the PEG-*b*-polypeptide into water. Moreover, UV-irradiation at 254 nm led to photo-crosslinking of the micellar core, which was directly proven by dynamic light scattering (DLS), showing a decreased size of the nanoparticle after core-crosslinking. Jing and coworkers reported the synthesis and ROP of a functional NCA monomer bearing a cinnamyl moiety [67]. Water soluble PEG-amine macroinitiator was utilized during the polymerization process, leading to well-defined PEG-*b*-polypeptide copolymers, which possess cinnamyl groups in the side chains of hydrophobic polypeptide (Figure 1A). The block copolymer was capable of self-assembling into micelles that could be core-crosslinked under UV light (Figure 1B,C). It is noteworthy to mention that Jing’s direct polymerization approach achieved full functionalization of cinnamyl in the repeating units of the polypeptide chain. However, in the case of Chen’s post-modification method, only a partial functionalization of repeating units with cinnamyl could be realized because of the low efficiency of esterification under steric environment of the polypeptide.

**Figure 2:**
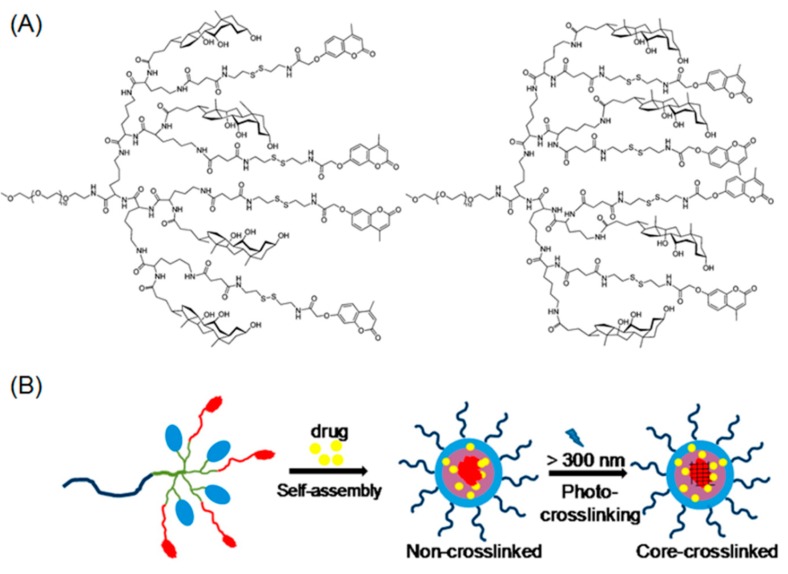
(**A**) Chemical structures of linear-dendritic PEG-*b*-polylysine containing coumarin moieties at their periphery; (**B**) light-triggered photo crosslinking of drug-loaded micelles. Reproduced with permission from [68].

Beyond cinnamyl photo-chemistry, coumarin based reversible dimerization has also been illustrated in the fabrication of photo-crosslinkable polypeptide micelles. In a pioneering work by Luo, solution-phase peptide synthesis (PPS) was employed to obtain a linear-dendritic block copolymer composed of hydrophilic PEG (5 KDa) and hydrophobic branched polylysine containing peripheral coumarin groups (Figure 2A) [68]. As the block copolymer was amphiphilic, micellar nanoparticles were observed in water as a result of self-assembly (Figure 2B). When long-wavelength UV irradiation (>310 nm) was applied to micelle solutions, the core-crosslinking event was rapidly completed within 400 seconds, as indicated by UV-Vis spectra. More interestingly, photo-induced decrosslinking occurred upon exposure to a short wavelength UV light (254 nm), elucidating the reversibility of this process. Notably, the decrosslinking reaction of coumarin dimer underwent a significantly slower kinetics (over 100 min) compared with that of crosslinking process.

### 2.2. Photo-Cleavable Nano-Objects

While polypeptide derived diblock copolymer micelles can acquire enhanced stability through the photo-crosslinking process, the concern regarding the lack of degradability still remains, especially in biomedical applications [86,87]. In view of this, photo-cleavage chemistry has emerged as an alternative approach to photo-sensitive polypeptide nanoparticles [13,69,70,71,72,74,88]. More importantly, photo-cleavage reactions are typically accompanied by a dramatic increase in the water-solubility of the hydrophobic segment, which could promote either a disassembly or morphological transformation of nano-objects. 

**Figure 3:**
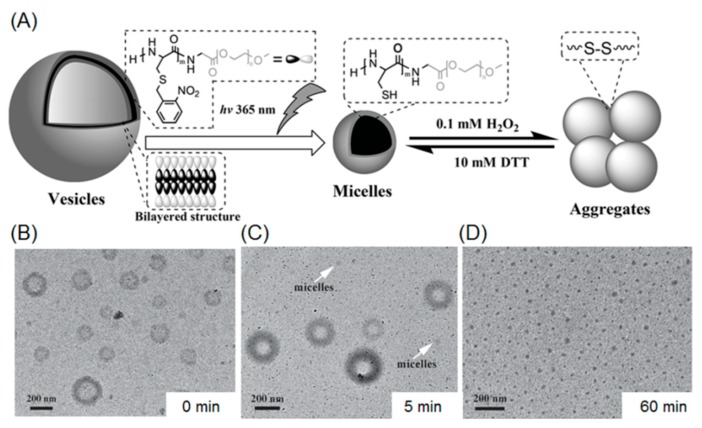
(**A**) Shape programming of polypeptide based nanoparticles through photo-regulation and redox process; (**B**) vesicular structures before UV treatment; (**C**) a mixture of vesicles and micelles after UV irradiation for 5 mintes; and (**D**) after UV-irradiation for 1 h, the vesicles were fully transformed to micelles. Reproduced with permission from [70].

Several illustrative examples of photo-cleavable polypeptide nanoparticles involving *O*-nitrobenzyl groups have been reported by Dong et al. In their first work, a photo-sensitive *S*-(*O*-nitrobenzyl)-l-cysteine NCA monomer (abbreviated as NBC) was designed and polymerized with PEG-amine as macroinitiator, giving rise to a diblock copolymer PNBC-*b*-PEG [69]. Since the NBC repeating units are hydrophobic because of the presense of *O*-nitrobenzyl moieties in the side chains, the amphiphilic block copolymer is able to form micelles with a size of 79 nm. This approach conferred photo-degradability to the micelles, because the hydrophobic core consists of numerous UV-labile *O*-nitrobenzyl groups. Transmission electron microscopy (TEM) and dynamic light scattering demonstrated that the block copolymer micelles were capable of dissociating into smaller nanoparticles (44 nm) upon UV irradiation at 365 nm. The reduction in particle size is because of the photo-cleavage of *O*-nitrobenzyl groups, producing free thiols with enhanced water solubility. A later report described the photo-induced shape transformation of polypeptide-containing vesicles (Figure 3A) [70]. In that work, PNBC_56_-*b*-PEG_114_ (the subscript stands for the number of repeating units) was synthesized and used for constructing a vesicle morphology in an aqueous solution. The vesicle solution was subsequently exposed to 365 nm UV light, promoting the cleavage of *O*-nitrobenzyl groups and a concomitant increment in hydrophilicity of PNBC block. As the ratio of hydrophilicity to hydrophobicity increased, the critical packing parameters of the nano-assemblies decreased, inducing a morphological transition from vesicle to micelles (Figure 3B–D). In addition, the free thiol inside the micellar core can be further oxidized in the presence of an oxidizer (i.e., hydrogen peroxide), resulting in formation of disulfide linkages, which prompt the aggregation of the micelles.

**Figure 4:**
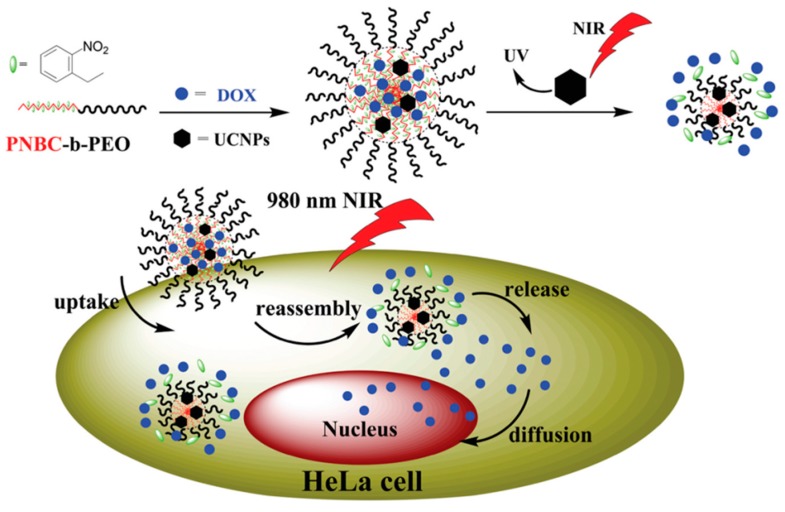
Fabrication of NIR-responsive polypeptide micelles via encapsulation of upconversion nanoparticles (UCNP) into block copolymer micellar core. Upon NIR irradiation, UCNP converts NIR into UV light, which further cleaves *O*-nitrobenzyl moieties and induces disassembly of micelles. Reproduced with permission from [13].

**Figure 5:**
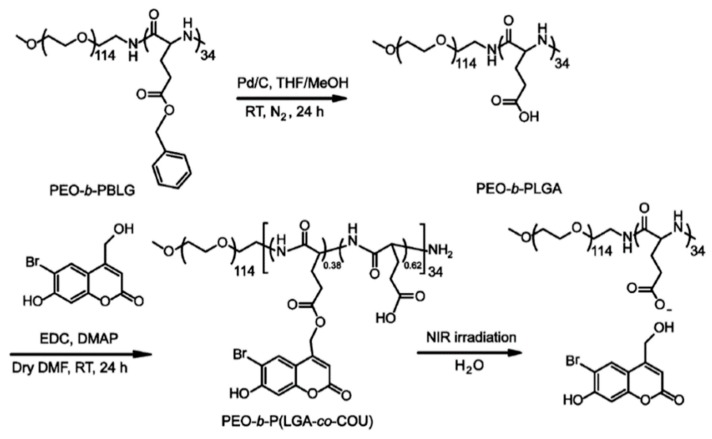
Synthetic route to NIR-responsive diblock copolymer consisting of PEG and polypeptide bearing coumarin groups. Reproduced with permission from [74].

Very recently, the same group invented NIR-responsive PNBC-*b*-PEG upconversion composite micelles (Figure 4) [13]. During the block copolymer self-assembly process, upconversion nanoparticles (UCNP) were encapsulated inside the PNBC core. The composite micelles were capable of disassembling with the help of UCNP, converting NIR light (980 nm) to UV light (365 nm). Moreover, Zhao and coworkers reported a novel NIR light-sensitive micellar system based on a diblock copolymer, consisting of PEG and poly(l-glutamic acid) bearing pendent 6-bromo-7-hydroxycoumarin-4-ylmethyl groups, an efficient NIR two-photon-absorbing chromophore (Figure 5) [74]. Upon irradiation with 794 nm of NIR light, the chromophores were gradually removed from the polypeptide chain, shifting the hydrophilic–hydrophobic balance toward a disassembly of micelles in water. Notably, nearly 200 min of irradiation was needed to fully cleave the side chain groups, demonstrating the potential of controlled release kinetics.

### 2.3. Photo-Isomerizable Nano-Assemblies

According to the properties of the aforementioned photo-crosslinkable and photo-cleavable polypeptide nanoparticles (vide supra), we can easily draw the conclusion that the photo-induced shape transformation or micellar disruption based on those functionalities are non-reversible under common conditions. While the de-crosslinking reaction of coumarin dimer can be literally achieved, the condition (i.e., 254 nm) is harsh and the slow reaction could cause the decomposition of coumarin and lead to undesired side reactions [89]. In the case of *O*-nitrobenzyl, the UV-induced photo-redox cleavage would generate *O*-nitrosobenzaldehyde that cannot reform the original *O*-nitrobenzyl moiety. To further pursue efficient and reversible photo-responsiveness of polypeptide nano-assemblies, some research groups have designed smart nanoparticle systems, which rely on photo-isomerizable functionalities, such as azobenzene and spiropyran [40,75,76,77,78,90,91,92].

**Figure 6:**
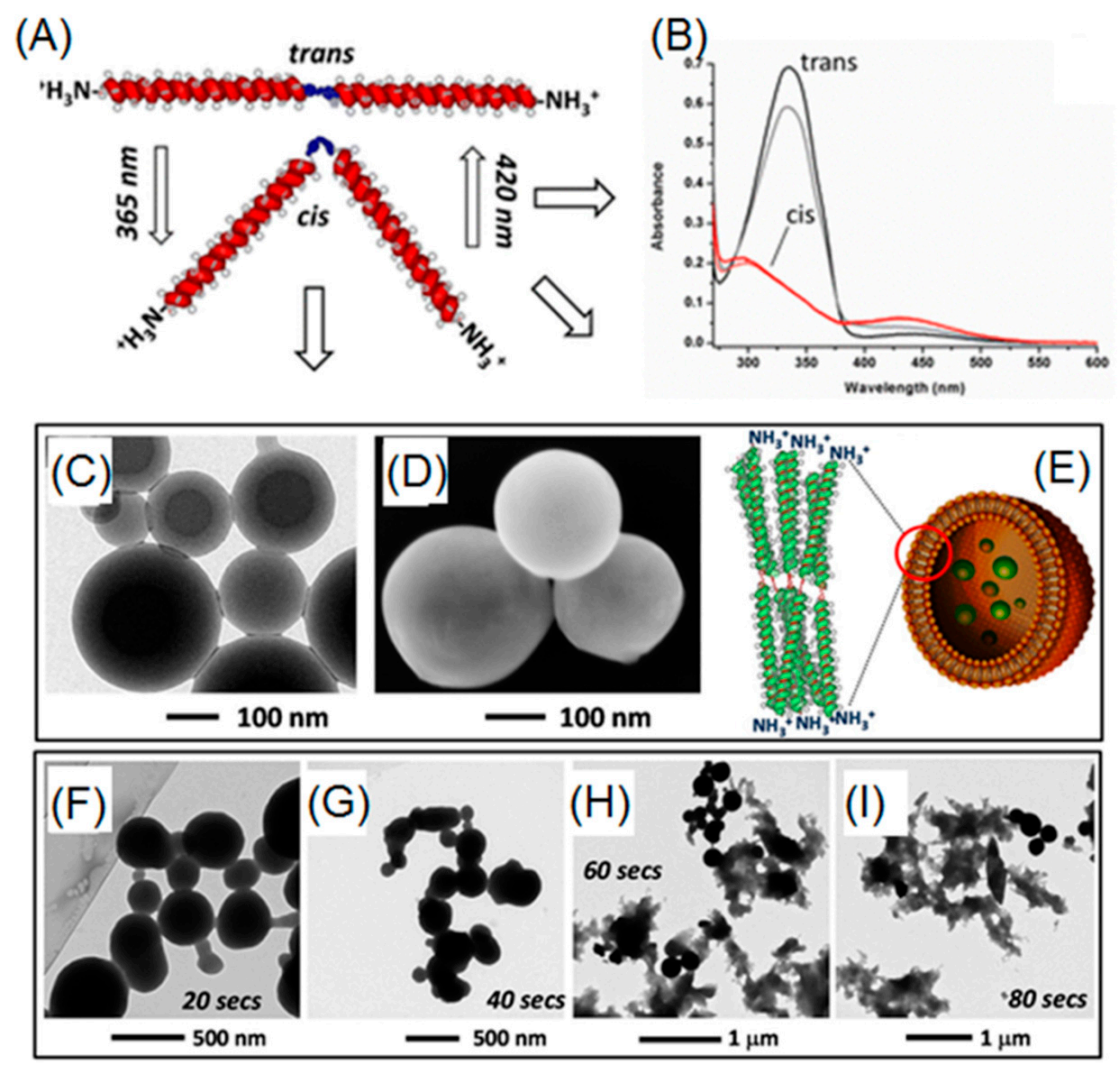
(**A**) Reversible geometry change of azobenzene-containing polypeptide via UV and visible light; (**B**) UV-Vis absorptions of *cis-* and *trans-*isomers; (**C**,**D**) TEM and SEM images of vesicles arising from self-assembly of *trans-*isomer of polypeptides; (**E**) cartoon representation of vesicular structure based on *trans-*polypeptide; and (**F**–**I**) time-dependent UV-induced degradation of vesicles. Reproduced with permission from [76].

Azobenzene is capable of transitioning between two isomers (i.e., *cis* and *trans*) through manipulation of UV light (365 nm) and visible light. When UV light is present, a polar *cis-*isomer is favorably formed. On the other hand, visible light or heat can promote the shift of isomerization towards thermodynamically favored non-polar *trans-*isomer. To date, azobenzene derivatives have been extensively incorporated into many peptides, either in the side chains or in the backbone. In a report by Moretto, azobenzene served as a central linker for diblock poly(*γ*-benzyl-l-glutamate) (PBLG) (Figure 6A,B) [76]. Before UV irradiation, diblock PBLG *trans-*isomer vesicles were observed, as evidenced by TEM and SEM (Figure 6C,D). After exposure to UV light, a rapid and gradual collapse of those ordered vesicles was observed, probably owing to *trans-*to-*cis* azobenzene transformation, which induced change in 3D geometry of diblock polypeptide (Figure 6F–I). Lu and coworkers were able to synthesize photo-responsive polypeptides via ROP of NCA monomers that consisted of pendent azobenzene and oligoethylene glycol (OEG), affording P(OEG-Azo) [40]. Because of the presence of both hydrophobic azobenzene and hydrophilic OEG, P(PEG-Azo) can self-assemble into nanoparticles in an aqueous solution. Moreover, a *α*-helical conformation of polypeptide was observed in the case of azobenzene *trans-*isomer. Upon UV treatments, *trans*-*cis* isomerization occurred and forced the polypeptides to adopt a disordered conformation, as evidenced by the circular dichroism spectroscopy. Importantly, a reversible conformation switch was found when heating the UV-treated *cis-*polypeptides at 70 °C.

**Figure 7:**
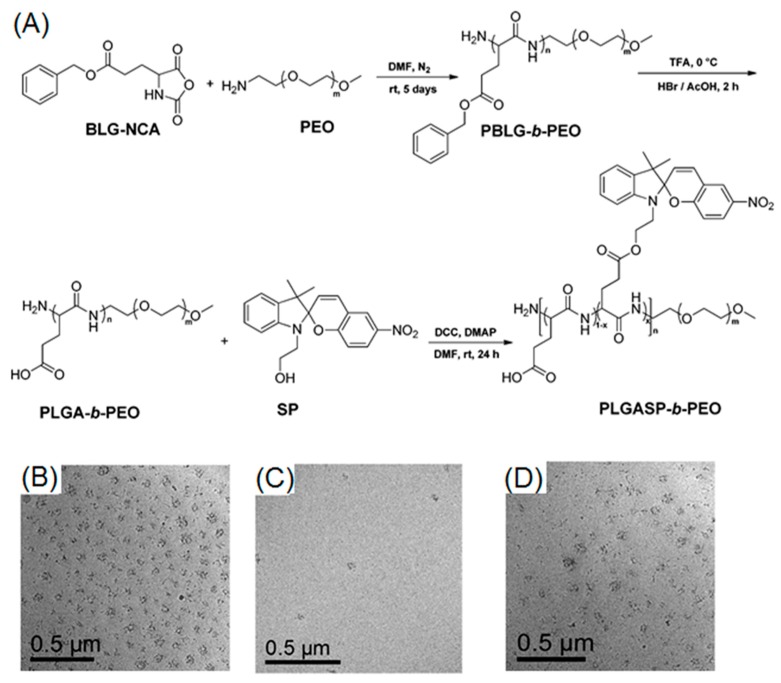
(**A**) Synthetic route to spiropyran-bearing polypeptide-*b*-PEG diblock copolymer; (**B**) TEM image of polymer nano-objects before UV treatment; (**C**) TEM image after UV irradiation; and (**D**) TEM image of regenerated micelles after applying visible light to UV-treated polymer solution. Reproduced with permission from [78].

Spiropyran (SP) is a widely-used photochromic molecule, thanks to light-induced spiropyran-to-merocyanine (SP–MC) isomerization [93,94]. Original SP derivatives, in their closed form, appear as colorless, nonpolar, and hydrophobic compounds. Isomerization toward MC (open form) occurred under UV treatment, leading to MC derivatives, which are colored, polar, and hydrophilic. Mezzenga et al. presented an excellent example of photo-reversible micelle system, based on spiropyran-containing polypeptide-*b*-PEG diblock copolymer (Figure 7A) [78]. Firstly, they performed a kinetic study of SP–MC and MC–SP isomerization, using UV-Vis spectroscopy. Before UV irradiation (365 nm), the solution was colorless, suggesting the absence of the MC form. After UV irradiation, the absorption peak at 544 nm progressively increased and reached maximum value within 5 min, indicative of fast and complete SP–MC isomerization. Nevertheless, MC–SP isomerization happened much slower and reached full conversion after 180 min in the presence of visible light (590 nm). After demonstrating the photo-regulated reversibility of SP–PC isomerization, the authors further utilized TEM to observe the reversible aggregation–dissolution–aggregation process of block copolymers in water. According to their results, original SP isomer containing polymers were capable of self-assembling into micelles (Figure 7B). UV irradiation fully disrupted the micellar structure after 5 min, because of the formation of hydrophilic MC moieties (Figure 7C). Interestingly, micelles were successfully recovered as a consequence of visible light treatment for 3 h (Figure 7D).

## 3. Properties and Applications

**Figure 8:**
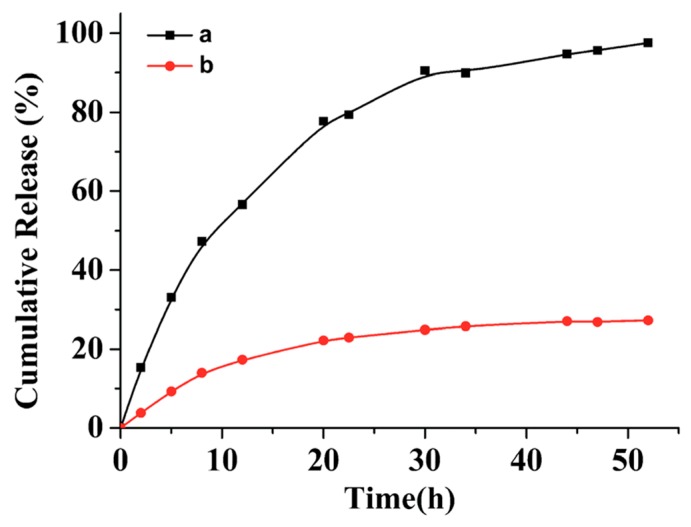
Cumulative release of drugs from non-crosslinked polypeptide micelle (black square) and photo-crosslinked polypeptide micelle (red dots). Reproduced with permission from [67].

Apparently, polypeptide derived nanoparticles hold great potential to serve as excellent drug delivery systems because of their biocompatibility and biodegradability. Moreover, the aforementioned photo-chemistry confers those nanoparticles with attractive properties, such as enhanced colloidal stability and on-demand drug release. Jing and coworkers investigated in vitro paclitaxel (PTX) release from two batches of PTX-loaded peptide micelles, with one batch treated with UV light [67]. According to their results, the drug release from the crosslinked micelles was significantly slower than that from the non-crosslinked micelle. For instance, only 20% of the drug was leaked from a crosslinked micelle during 55 h incubation in a phosphate buffered saline (PBS) buffer, while almost 100% of the drug was released from a non-crosslinked micelle under same condition (Figure 8). In Zhao’s study, NIR-responsive Rifampicin-encapsulated polypeptide micelles showed a neglectable release after 55 h in the absence of NIR irradiation. When the NIR laser was turned on, a progressive drug release was observed, demonstrating the feasibility of this drug delivery system to achieve on-demand drug release.

**Figure 9:**
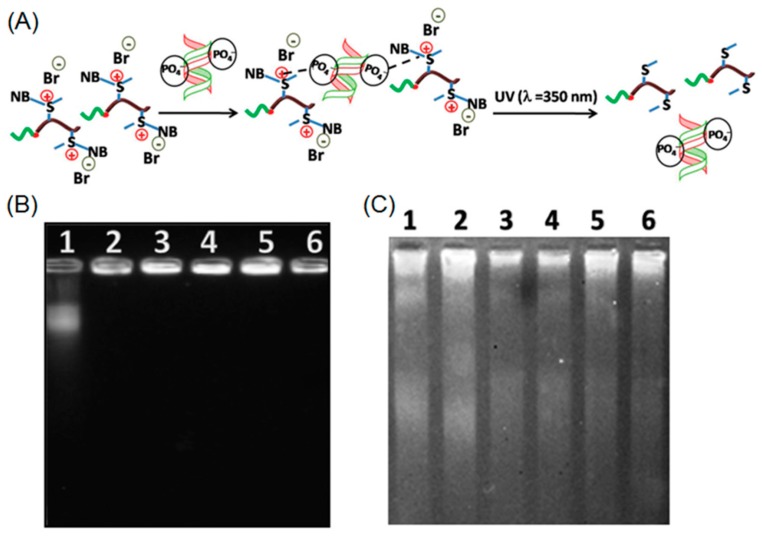
(**A**) Schematic illustration of polypeptide-DNA complexation and UV-induced release of DNA; (**B**) electrophoretic mobility of polypeptide-DNA conjugates at different concentrations of cationic polypeptide before UV irradiation; and (**C**) electrophoretic mobility of polypeptide-DNA conjugates upon UV treatment. Note: lanes 1–6 correspond to 12.7 mM ctDNA complexing with polypeptide at 0, 0.2, 0.4, 0.6, 0.8, and 1.0 mM, respectively. Reproduced with permission from [73].

Very recently, Mandal and coworkers employed ROP to prepare a cationic block copolymer consisting of poly(2-ethyl-2-oxazoline) and positively charged *O*-nitrobenzyl modified polymethionine (P[MetNB][Br]) [73]. This cationic polypeptide was capable of forming an electrostatic complex with negatively charged calf thymus DNA (ctDNA). When UV-light was applied to the polypeptide-DNA complex, a photo-driven cleavage of *O*-nitrobenzyl moieties occurred, resulting in neutral polypeptides, which had no binding affinity with ctDNA (Figure 9A). According to the gel electrophoresis, free ctDNA was rapidly released from the complex after irradiation with UV light. Those results demonstrated the potential of using photo-responsive cationic polypeptide as a DNA delivery platform (Figure 9B,C).

**Figure 10:**
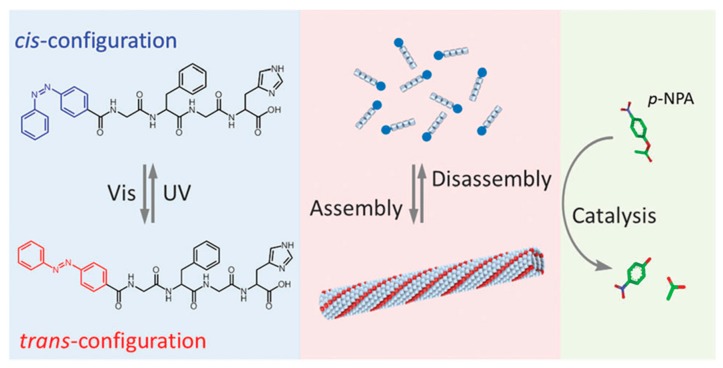
Molecular structures of azobenzene-terminated peptide and photo-switchable assembly and catalytic activities of the peptide, based artificial hydrolase. Reproduced with permission from [77].

In addition to biomedical applications, photo-responsive polypeptides have been used in the field of catalysis as well. He and coworkers designed a peptide-based artificial hydrolase, which consisted of a catalytic histidine residue and a photo-responsive azobenzene group in the peptide chain (Figure 10) [77]. Before UV irradiation, the peptide exhibited an antiparallel *β*-sheet conformation, enabling self-assembly into a peptide fibril. An enhanced catalytic activity on *p*-nitrophenyl acetate was observed, because of the hydrophobic environment of peptide fibril and proximity effect of histidine groups. However, a significant reduction in catalytic efficiency occurred upon exposure to UV-light, which caused a conformational conversion of peptide from *β*-sheet to random coil and thus disrupted the supramolecular fibril structure. Most importantly, the authors were able to demonstrate that the activity of the peptide-based artificial hydrolase could be reversibly controlled using visible and UV light.

**Figure 11:**
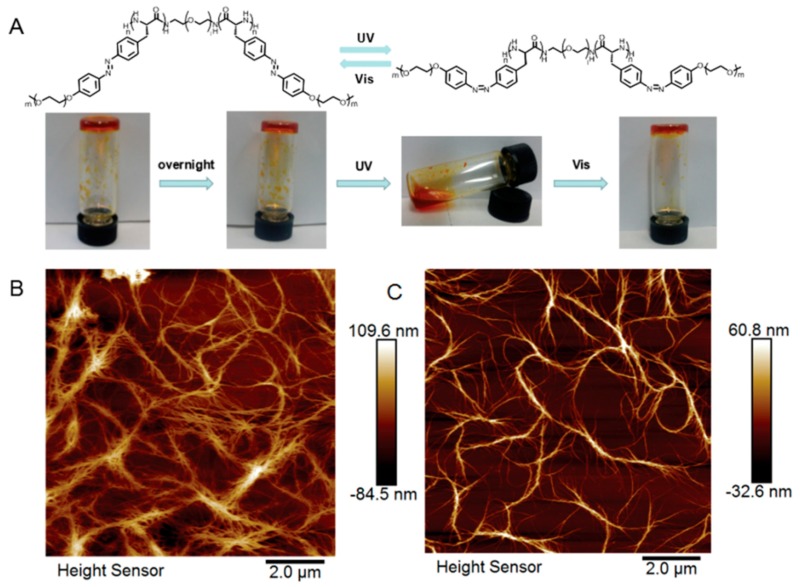
(**A**) Reversible sol-gel process by switching light wavelength between UV and visible; (**B**) atomic force microscopy (AFM) image of polymer solution before UV treatment; and (**C**) AFM image of polymer solution after UV irradiation. Reproduced with permission from [40].

Finally, the application of photo-sensitive polypeptides was successfully translated into macroscopic materials involving reversible sol-gel process, as described by Hu and Li (Figure 11) [40]. In their study, an organogel was formulated by dissolving azo-bearing polypeptide-*b*-PEG-*b*-polypeptide triblock copolymer in THF (Figure 11A). Interestingly, the gel was capable of switching physical states between gel and solution upon alternating the visible and UV treatment. According to the atom force microscope images, the gel revealed a densely crosslinked fibrous network, while the solution exhibited a much smaller degree of crosslinking after UV irradiation (Figure 11B,C).

## 4. Current Challenges and Prospective

Many relatively recent developments in photo-responsive polypeptides have greatly expanded the scope of smart nanomaterials, providing us with many new possibilities and opportunities in various applications, such as drug delivery, self-healing materials, and catalysis. Indeed, the marriage of polypeptide and photo-chemistry not only confer biocompatibility to the nanomaterials, but also facilitate the structural control of peptide chains or nano-assemblies because of the ease of using light. In view of photo-chemistry relying on different light-sensitive functionalities, a number of photo-sensitive peptide nanoparticles with distinct properties have been accomplished. 

Despite the tremendous success that has been described above, many challenges still remain. One significant barrier is the translation of light-responsive polypeptide drug delivery system into clinical use. Indeed, the majority of examples in this review involve the use of UV light or visible light, which has a poor penetration depth into human tissue. Moreover, UV light has been shown to be detrimental to healthy cells and tissues [95,96,97,98,99]. Because of these downsides of using UV/Vis light, NIR-responsive polypeptide nanoparticles represent a more promising platform for nanomedicine [100]. However, the current NIR-responsive polypeptide derived drug delivery systems suffer from either slow drug release kinetics or an introduction of cytotoxic UCNP [13,74]. Therefore, more careful design and study are essential in order to translate those nanomaterials into biomedical applications. Moreover, photo-responsive polypeptide nano-objects have not yet been reported by means of controlled radical polymerization (CRP) and ring-opening metathesis polymerization (ROMP). Considering the robustness of CRP and ROMP techniques to prepare polymers with complex architectures and functions, we envision that one of the next directions for photo-responsive polypeptides will be updating the synthetic toolbox, in order to achieve more sophisticated polypeptide structures. According to the above-mentioned examples illustrated in Table 1, photo-responsive polypeptide deriving nanomaterials have been overwhelmingly exploited for potential biomedicine use, such as drug delivery and gene release. However, only a few reports demonstrated the promise of those materials in other utilities, such as switchable catalysis and reversible macroscopic gel materials. Since light can be easily used to dictate when and where the photo-reaction happens, it can be anticipated that the significant attention on photo-responsive polypeptides will be shifted to some other applications, including self-healing materials, lithography or 3D-printing technology, which may exhibit excellent performance with the help of light-stimulus. Given the considerable success of traditional stimuli-responsive materials in biomedicine and manufacturing, we believe that photo-responsive polypeptide nanomaterials will take on more important roles to next generation of supramolecular peptide nanotechnology and material science.

## Figures and Tables

**Table 1:** Summary of photo-responsive polypeptide nano-assemblies.

Polypeptide	Synthetic Method	Photo-Responsive Moiety	Light-Triggered Events	Application	Ref.
PEG-*b*-P(LGA/CLG)	ROP *^a^*	Cinamyl	Micellar Core-Crosslink	Drug Delivery	[66]
PEG-*b*-PCLG	ROP	Cinamyl	Micellar Core-Crosslink	Drug Delivery	[67]
PEG-CA_4_LS_4_Co_4_	Solution-PPS *^b^*	Coumarin	Micellar Core-Crosslink	Drug Delivery	[68]
PNBC-*b*-PEO	ROP	*O*-nitrobenzyl	Micellar Disassembly	Drug Delivery	[69]
PNBC-*b*-PEO	ROP	*O*-nitrobenzyl	Vesicle to Micelle	N/A	[70]
PNBC-*b*-PEO	ROP	*O*-nitrobenzyl	Composite Nanoparticle	Drug Delivery	[13]
PNBL-*b*-PLL	ROP	*O*-nitrobenzyl	Sol-Gel Transition	N/A	[71]
PNBL-*b*-PEO	ROP	*O*-nitrobenzyl	Sol-Gel Transition	N/A	[72]
PEtOx-*b*-P[MetNB][Br]	ROP	*O*-nitrobenzyl	Composite Nanoparticle	Gene Delivery	[73]
PEO-*b*-P(LGA-*co*-COU)	ROP	6-Bromo-7-hydroxyl-coumarine	Micellar Disassembly	Drug Delivery	[74]
Fmoc-Phe-pazoDbg	Solution-PPS	Azobenzene	Micelle to Fiber	N/A	[75]
PBLG-azobenzene	ROP	Azobenzene	Vesicle Disruption	Cargo Release	[76]
P(OEG-Azo)	ROP	Azobenzene	Sol-Gel Transition	N/A	[40]
Azo-GFGH	Solid-PPS	Azobenzene	Nanofiber Assembly	Catalysis	[77]
PLGASP-*b*-PEO	ROP	Spiropyran	Micellar Disassembly	Drug Delivery	[78]

Note: *^a^* ROP—ring-opening polymerization; *^b^* PPS—phase peptide synthesis.
